# Extracted Compounds from Neem Leaves as Antimicrobial Agent on the Physico-Chemical Properties of Seaweed-Based Biopolymer Films

**DOI:** 10.3390/polym12051119

**Published:** 2020-05-14

**Authors:** U. Seeta Uthaya Kumar, S. N. Abdulmadjid, N. G. Olaiya, A. A. Amirul, S. Rizal, A. A. Rahman, Tata Alfatah, E. M. Mistar, H. P. S. Abdul Khalil

**Affiliations:** 1 School of Industrial Technology, Universiti Sains Malaysia, Penang 11800, Malaysia; 2 Department of Physics, Faculty of Mathematics and Natural Sciences Universitas Syiah Kuala, Banda Aceh 23111, Indonesia; 3 Department of Industrial and Production Engineering, Federal University of Technology, PMB 704 Akure, Ondo State, Nigeria; ngolaiya@futa.edu.ng; 4 School of Biological Sciences, Universiti Sains Malaysia, Penang 11800, Malaysia; amirul@usm.my; 5 Department of Mechanical Engineering, Universitas Syiah Kuala, Banda Aceh 23111, Indonesia; samsul_r@yahoo.com; 6 School of Physics, Universiti Sains Malaysia, Pulau Pinang 18000, Malaysia; arazhar@usm.my; 7 Environment and Forestry Office of the Provincial Government of Aceh, Banda Aceh 23239, Indonesia; tataalfatah83@gmail.com; 8 Chemical Engineering Department, Universitas Serambi Mekkah, Tgk. Imum Lueng Bata Street, Batoh, Banda Aceh 23249, Indonesia; eka.marya.mistar@serambimekkah.ac.id

**Keywords:** Azadirachta indica, seaweed, antimicrobial, biodegradable, composite, biopolymer

## Abstract

Neem leaves extract was incorporated into the matrix of seaweed biopolymer, and the seaweed-neem biocomposite films were irradiated with various doses of gamma irradiation (0.5, 1.5, 2.5, 3.5, and 4.5 kGy). The physical, barrier, antimicrobial, and mechanical properties of the films were studied. The incorporation of 5% *w*/*w* neem leaves extract into a seaweed-based film, and gamma irradiation dose of 2.5 kGy was most effective for improved properties of the film. The results showed that the interfacial interaction of the seaweed-neem improved with physical changes in colour and opacity. The water solubility, moisture content, and water vapour permeability and biodegradability rate of the film reduced. The contact angle values increased, which was interpreted as improved hydrophobicity. The tensile strength and modulus of the films increased, while the elongation of the composite films decreased compared to the control film. The film’s antimicrobial activities against bacteria were improved. Thus, neem leaves extract in combination with the application of gamma irradiation enhanced the performance properties of the film that has potential as packaging material.

## 1. Introduction

Biodegradable composite films from renewable sources as an alternative to synthetic plastic packaging have been studied to overcome the plastic degradation problem and subsequently to reduce the accumulation of plastic waste [1]. The accumulation of plastic waste has been considered as one of the major environmental global issues. Petroleum-based synthetic plastic packaging materials have been reported as the lead source of plastic waste due to their nonbiodegradable nature. Green manufacturing of plastic products has been proposed to solve the problem of plastic waste pollution. Natural polymers such as proteins and polysaccharides have been widely used in the development of biodegradable composite films. Biopolymers, through various production techniques, have been transformed into biodegradable films [2,3].

The material used for packaging applications has been reported to have resistance to rupture and abrasion to protect the packaged product, while maintaining its flexibility to adapt to the eventual deformations of the product [4]. The most important functional properties of plastic films have been described as their optical, mechanical, and barrier properties [2]. Seaweed extract biopolymer has the excellent film-forming ability and has unique properties such as good oxygen barrier, biodegradability, and nontoxic properties. These properties have attracted researchers for the possible use of seaweed as a biodegradable packaging film. Biodegradable films produced from seaweed have been reported to have low mechanical properties and water absorption [5]. Seaweed has been reinforced with other natural polymers with different preparation methods to produce seaweed-based films with improved mechanical and water absorption properties [6]. These studies were conducted with unaltered biodegradable properties. To further improve the mechanical and water absorption properties of biopolymers, researchers have proposed the use of a physical method such as ultraviolet and gamma irradiation, as one of the methods of modification of bio-based materials [7].

Gamma irradiation has been described as a physical method used for the enhancement of the properties of polysaccharides [2,5]. The application of gamma irradiation to biodegradable films has been reported to alter the characteristics and subsequently improved the mechanical and barrier properties of the films [6]. It was necessary to irradiate biodegradable films to induce a possible crosslink of the macromolecules as reported in the literature [8,9,10]. It must, however, be noted that the use of gamma irradiation on a packaged product or material has no hazardous effect [11,12,13].

Several researchers have reviewed and worked on antimicrobial systems, both nanometals and biobased materials [14,15,16]. Seaweed-based films have been used as antioxidants and antimicrobial packaging. A biopolymer with antimicrobial nanomaterials or bio-based materials has been expanded extensively in various applications such as food packaging and biomedical devices because of their intrinsic microbicidal activities [17]. Silver-based nano compounds have been the most frequently used commercial nanomaterial [18] because of their potent antimicrobial activities [15]. Films incorporated with a bio-based material such as plant extracts have been reported and proposed as packaging materials. These films were described to have excellent antioxidant and antimicrobial activity [19] because plants have been reported to be rich in a wide variety of secondary metabolites with antimicrobial properties such as tannins, alkaloids, flavonoids, and phenolic compounds [20]. Bioactive plant extracts, such as neem (Azadirachta indica) extracts or essential oils have shown effective antimicrobial activities against strains of bacterial pathogens [21].

Previous research on the antimicrobial activity of neem extracts showed that the incorporation of neem leaves extract as an antimicrobial agent in seaweed-based films enhanced the antimicrobial activities and mechanical properties of the films [22]. It was also demonstrated in the study, that the incorporation of neem leaves extracts at 5% *w*/*w* showed the most optimum and desired improvement in mechanical, water vapour permeability, and antimicrobial properties. Hence, this current study analyzed the physical, barrier, mechanical, optical, microscopic, and antimicrobial properties of gamma-irradiated seaweed-neem biodegradable films for possible use as packaging material.

## 2. Materials and Methods

### 2.1. Materials

Ethanol (95% pure) of vapour density 1.59, boiling point 78.3 °C, melting point −114 °C, and density of 0.789 g/mL at 20 °C was purchased from Sigma-Aldrich Co., St. Louis, MO, USA. Moreover, the glycerol was purchased from Sigma-Aldrich Co., St. Louis, MO, USA with a boiling temperature of 182 °C/20 mmHg, melting point 20 °C, and density of 1.25 g/mL was used for this study. The seaweed was purchased from Green Leaf Synergy Sdn. Bhd., Tawau, Sabah, East-Malaysia), while the neem leaves were picked from Bukit Mertajam, Penang, Malaysia.

### 2.2. Preparation of Neem Leaves Powder

The fully matured Azadirachta indica (neem) leaves were collected and washed under tap water to remove any dirt. The leaves were air dried for 20 days, then ground into a powder and stored in airtight bottles.

### 2.3. Extraction of Neem Leaves

Ethanol was used for the isolation of neem leaves extract from its leaves as described by Al-Hashemi [23] with slight modification. The neem leave powder (170 g) was soaked in 95% ethanol (1700 mL) in a covered 1 L conical flask and placed in a reciprocating shaker at 28 °C for 24 h. The whole extract was filtered with a Whatman No. 42 (125 mm) filter paper and cotton wool. The obtained filtrate solution was concentrated, and ethanol was evaporated in a vacuumed rotary evaporator (Buchi Rotavapor R-200, Daya Qurban Saintifik Sdn Bhd., Flawil, Switzerland) with the hot water bath set at 40 °C to form a paste. The extracted paste was dried further in the oven at 40 °C to get a thicker paste (47.27 g). The crude neem extract obtained was sealed in a Petri plate and at room temperature (28 °C). The percentage of crude ethanol extract yield was calculated based on the weight of dried and ground neem leaves.

### 2.4. Preparation of Seaweed-Neem Films

The fabrication of seaweed-neem films was prepared with the method reported by Shellikeri et al. [24]. Seaweed (1%, w/v) with the weight of 6 g was dissolved in 300 mL distilled water at 82 °C and stirred mechanically for 15 min. An amount of 1.8 g of glycerol (50% *w*/*w* seaweed) was added and stirred for another 25 min. The neem leaves extract of 5% *w*/*w* was added to the seaweed film solution, based on previous work on the incorporation of different neem leaves extract in the seaweed-based film [22] and cast on a Teflon-coated plate. The cast solution mix was then dried in an oven at 40 °C for 24 h and stored in a desiccator at a relative humidity of 53% for 72 h for analysis. The films after 72 h were irradiated with different radiation doses of 0.5, 1.5, 2.5, 3.5, and 4.5 kGy with a ^60^Co gamma-radiation source at room temperature. The evaluation of the physical, mechanical, barrier, and antimicrobial characteristics of the irradiated seaweed-neem films were conducted and compared to the nonirradiated seaweed-neem film (control film).

### 2.5. Characterization of Seaweed-Based Films Incorporated with Neem Leaves Extract and Irradiated

#### 2.5.1. Fourier Transform Infrared Spectroscopy (FT-IR)

FT-IR analysis of the films was studied with a Spectrum 8900 IR Spectrometer (Shimadzu, Chiyoda-ku, Tokyo, Japan) in the zinc selenide attenuated total refraction method (ATR) cell in an infrared spectrometer. The films were cut into a square shape with a dimension of 10 mm × 10 mm and then oven dried at 60 °C overnight. The measurement of the spectra was done in transmittance mode and wavenumber range of 400 to 4000 cm^−1^.

#### 2.5.2. Surface Characterization by Scanning Electron Microscope (SEM) and Atomic Force Microscope (AFM)

The film surface morphology was observed with SEM, Zeiss EVO MA10 (Carl Zeiss, Oberkochen, Germany). The samples were prepared with a gold-palladium coat to improve the conductivity for the SEM electron beam. The samples were coated with a Polaron (Fisons) SC515 sputter coater (Fison Instruments, VG Microtech, Sussex, UK). The samples were held in position on the holder with a carbon adhesive tape, and the SEM images were captured at 100 magnification to observe the effect of the irradiation, as well as the miscibility of the composite film. The AFM (XE-70 Park System, Park System, Suwon, Korea) was used to measure the surface roughness of the films. The film samples (2 cm × 2 cm) were placed on freshly cleaved mica and analyzed at a 0.3 Hz scan rate. The 3D images from the analysis were obtained at a set point of 14 nm and scan area of 30 μm.

#### 2.5.3. Colour and Opacity Properties

The physical appearance (colour and opacity) of the films was observed with the method proposed by Abdul Khalil et al. [1]. The Data Colour 400 Bench-Top Spectrophotometer was used with the Data Colour Match software (Data colour International, Lawrenceville, GA, USA) based on the CIE L*a*b* colour scale. The coordinates were obtained with the software. Where L* describes lightness (from black to white), a* and b* describe the chromatic coordinates (from −*a*: Greenness, −*b*: Blueness, +*a*: Redness, +*b*: Yellowness). The film samples were read on the surface of the standard white background as L* = 93.42, a* = −1.14, b* = 5.01. The measurements were replicated three times to obtain a mean result per sample. The colour difference (∆*E*) was determined based on Equation (1)

(1) $$\Delta E=\sqrt{{\left(L^{*}-L\right)}^{2}+{\left(a^{*}-a\right)}^{2}+{\left(b^{*}-b\right)}^{2}}$$

The difference in percentage in the contrast of the samples to standard white and black was calculated and recorded as the film opacity. This was done for three repeated samples, and the average was calculated.

#### 2.5.4. Moisture Content

The moisture content of the film was calculated with the difference in weight of the samples. The samples were cut into 10 × 10 mm and placed in an oven for 24 h at 105 °C. The initial weight, *M_i_* before heating, and the final weight *M_f_* after heating for 24 h were measured. Five samples were tested for each percentage composition variation, and the average values were reported. Moreover, the moisture content was calculated with Equation (2).

(2) $$Moisture\;content\;\left(\%\right)=\frac{M_{i}-M_{f}}{M_{i}}\times 100$$

#### 2.5.5. Solubility

Water solubility (WS) was studied with the method of Jafarzadeh, Alias [11]. Samples were cut to standard sizes (30 mm × 30 mm) and conditioned in a desiccator for three days in a silica gel. The samples were weighed, placed in 80 mL of distilled water, and agitated at 100 rpm for 1 h at room temperature. The film leftover was filtered with filter paper and dried at 60 °C in an oven until constant weight. The solubility of the films was calculated according to Equation (3) with *W_i_* as the initial dry weight and *W_f_* the final dried weight of the film.

(3) $$Water\;solubility\;\left(\%\right)=\frac{W_{i\;}-W_{f}}{W_{f}}\times 10$$

#### 2.5.6. Water Vapour Permeability (WVP)

The water vapour permeability (*WVP*) was determined at ASTM E96 [12]. A 5 cm diameter sealed glass beaker was used, and this was filled with 20 mL of distilled water. The samples were weighed and then placed in the beaker covered up with water and placed under a controlled chamber at 25 ± 2 °C and 50% ± 5% RH. Twelve sealed beakers were used, and the record of the setup beakers was taken each hour for 6 h. A graph of weight change against time was plotted for each sample to obtain the water vapour transmission rate (*WVTR*) and Equation (4) was used to calculate *WVP* for the films.

(4) $$WVP=\frac{WVTR\times L}{\Delta P}$$

The *WVTR* was measured in g/(m^2^ s), the mean film thickness (*L*) in meters (m), and the difference in water vapour pressure across the sides of the film (Δ*P*) in Pa.

#### 2.5.7. Wettability Test

The contact angle was studied with KSV CAM 10 (KSV Instruments Ltd., Espoo, Finland) to determine the wettability of the film. The sample was placed in the instrument, and the snap of the test liquid dropped at a speed of 5 fps on the surface of the film was recorded for 5 s. This was done for five repeated samples, and the average calculated.

#### 2.5.8. Tensile Properties

The Miniature Tensile Tester MT1175 (Dia-Stron Limited Instruments, Andover, UK) was used to determine the tensile properties of the film. The tensile tester was programmed with the UvWin1000 software (Dia-Stron Limited Instruments, Andover, UK), and the ASTM D882-12 [25] standard was used. Samples were cut off regularly with a length of 150 mm and a width of 5 mm. A digital micrometer was used to determine the thickness. The thickness was calculated by the arithmetic mean of three random measurements in different segments of the film. The value of the tensile strength and elongation at break was calculated as the average of five measurements of each film sample. The tensile strength, tensile modulus, and elongation were obtained.

#### 2.5.9. Antimicrobial Activity

The antimicrobial activity was studied with three food pathogens. The selected pathogens for the test were *Escherichia coli, Staphylococcus aureus,* and *Bacillus subtilis.* The samples bacterial of 10^7^ CFU/mL were cultured with a sterile solution at 37 °C for 24 h. Punched film of a 6 mm disk diameter was used for the test, and the agar diffusion assay was used to determine the antimicrobial activities. The bacterial were cultured overnight with inoculated agar plates. They were later incubated at 37 °C for 24 h. A calliper was used to measure the diameter of the inhibition zone and indicated by mm as the diameter of the zone. Analyses were conducted in triplicate.

#### 2.5.10. Soil Burial Test

Composite film degradation was determined by weight loss during soil burial, according to Seligra, Jaramillo [19] with adjustment. The test was setup with samples (3 cm × 3 cm) buried at ambient temperature and 4 cm below the soil with approximately 36% (*w*/*w*) moisture content. The degradation weights were measured after 0, 10, 20, and 30 days. The percentage of weight loss was calculated with Equation (5).

(5) $$Weight\;loss\left(\%\right)=\frac{W_{1}-W_{f}}{W_{1}}\times 100$$

*W*_1_ = initial dry weight of composite film

*W*_2_ = dry weight of composite film after cleaning

## 3. Results and Discussion

### 3.1. Fourier Transform Infrared Spectroscopy (FT-IR)

**Figure 1:**
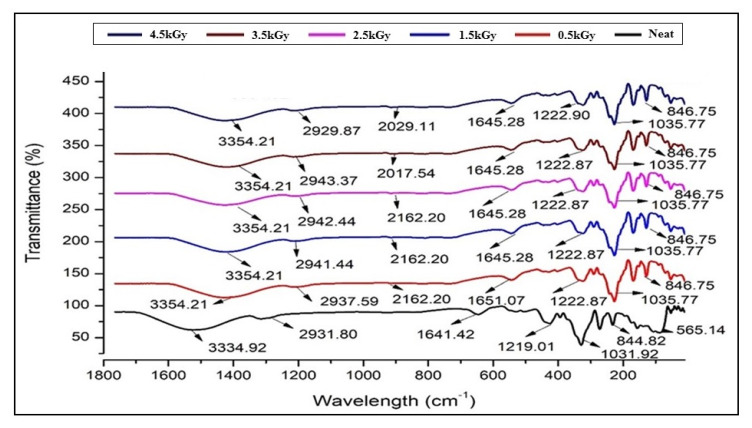
FT-IR spectra of seaweed-neem biocomposite films with different radiation doses (0.5, 1.5, 2.5, 3.5, and 4.5 kGy).

Figure 1 showed the FT-IR data for control and irradiated seaweed-neem composite films. In general, all the spectra of the films showed a similar pattern of bands. The bands at approximately 3100–3700 cm^−1^ were assigned to the hydrogen-bonded hydroxyl group (OH) from the complex vibration stretch, associated with free, inter, and intramolecular bound hydroxyl groups. The FT-IR spectra showed absorption bands in the 1000 to 1200 cm^−1^ region that correspond to the c-o stretch and 925 to 935 cm^−1^ regions, due to the C–O in 3,6-anhydrous-d-galactose. When the film was irradiated, the peak positions shifted compared to the control films, while the fingerprint peak absorption of amide ii (1641.4 cm^−1^) and –CH_3_ (2931.8 cm^−1^) bend vibration shifted toward a higher frequency of 1645.3 and 2939.5 cm^−1^, respectively. The spectra of the irradiated seaweed-neem films showed a new peak at 2000 to 2500 cm^−1^ compared to the control film, which was associated with the absorption stimulated by triple bonds. The infrared spectrum of seaweed-neem films with various gamma-irradiation doses (0.5–4.5 kGy) showed almost similar peaks with the nonirradiated film and with one additional new functional group formed. It was found that the peaks of the irradiated films were sharper and showed differences in peak intensity as compared with control films. In conclusion, their formation of one new functional group indicated that the interaction between seaweed matrix and neem leaves molecules occurred with bonds decomposition by irradiation.

### 3.2. Surface Characterization of Seaweed-Neem Films

**Figure 2:**
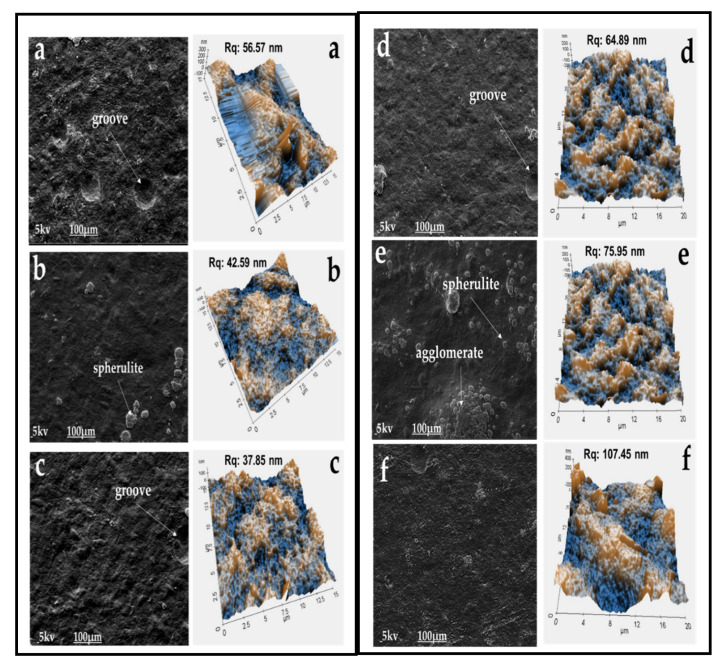
The surface morphologies by scanning electron microscope (SEM) and surface roughness by atomic force microscope (AFM) of irradiated seaweed-neem films (**a**) 0.5, (**b**) 1.5, (**c**) 2.5, (**d**) 3.5, (**e**) 4.5 kGy and (**f**) control film (non-irradiated), at 1000 times magnification.

The surface morphology of the seaweed-neem films with various doses of radiation was observed by SEM analysis, and the surface roughness (R_q_) was detected by AFM. The micrographs of the film surface were presented in Figure 2. The surface of the control film was rough, with the Rq value of 107.45 nm after the addition of neem leaves extract to the seaweed matrix in film formation. At the same time, the SEM image of irradiated seaweed-neem composite films showed a smoother surface and lower R_q_ value. From Figure 2, it was concluded that the increase in irradiation doses had reduced the surface roughness of seaweed-neem films. This effect was seen at an irradiation dosage of 2.5 kGy than at higher dosages (3.5 and 4.5 KGy) with the R_q_ value of 37.85 nm, as shown in Figure 2. The seaweed-neem films after radiation application indicated a clear change of surface morphology due to the physical modification by irradiation. The results obtained reflected the changes in seaweed structure, such as aggregate formation and the variance in aggregate size and configuration. This study was in agreement with Rahman, Dafader [26] on the microstructure of irradiated alginate-starch-citric acid blend films with a smoother surface than the control film. The changes in the microstructure of the films in this study could be due to the change in the surface morphology of the blend film by radiation.

### 3.3. Colour and Opacity

Colour has been reported as an important property for materials used for packaging. The colour value was recorded as L* (lightness, 0 = black, 100 = white), a* (−a* = greenness, +a* = redness), and b* (−b* = blueness, +b* = yellowness). The colour measurement of the films in Table 1 reflected the chromatic coordinates of control and irradiated seaweed-neem films. The results depicted that the L* value was increased, indicating enhanced brightness as the radiation dose increased up to 2.5 kGy. At the dose of 0.5 to 2.5 kGy, the values of L* increased, and the highest value was presented at the dose of 2.5 kGy. The control seaweed-neem film without irradiation had a lower value of b*, while the irradiated films had higher values of b*. The lowest value of b* was presented at a dose of 2.5 kGy. Generally, gamma radiation notoriously intensified the yellowish tone of the film. Moreover, irradiated films had higher values (greener), and the film with a 2.5 kGy radiation dose showed the highest value of a* and greenness. These results were similar to the results obtained from the previous report, which showed that the yellowness of films was increased by radiation [6,27]. The change in the film colour was probably due to the chemical and biological changes of the molecule. Seaweed-based films showed a darker colour because of the chlorophyll and carotenoid pigment in the seaweed, which gives a green colour to the seaweed-neem films [28].

Opacity was measured to determine the relative transparency of the film. A high value of opacity indicated that the specimens have low transparency or a more opaque nature [29]. Opacity measurements of the films have been described as important properties of films used as packaging materials. The measurement was necessary for this study because it controls the penetration of sunlight, fluorescent light, or incandescent light across the films due to photodegradation [28]. In some film packaging, protection against incident light was required, especially for products with sensitive light-catalyzed degradation reactions [30]. In others, transparency was desirable for good visual presentation of the packaged product to consumers. In visual observation, seaweed-neem films remain transparent after irradiation at doses up to 10 kGy. The values of the opacity of the seaweed-neem films were presented in Table 1.

The opacity of the control seaweed-neem film was 17.02 ± 0.007. The different doses of irradiation significantly influenced (*p* < 0.05) the opacity of the films. The seaweed-neem film irradiated with 2.5 kGy exhibited the lowest opacity, 16.56 ± 0.008 compared to the other composite films. The opacity of the composite films decreased gradually as the doses of radiation increased up to 2.5 kGy, indicating an increase in the film transparency. However, the irradiation dose of 3.5 and 4.5 kGy exhibited an increase in film opacity, indicating a decrease in film transparency. The fluctuations of the opacity of the composite films were in a narrow range, indicating that the transparency was not so much influenced by the irradiation absorbed doses in the range of 0.5–4.5 kGy.

### 3.4. Moisture Content and Water Solubility of the Films

**Figure 3:**
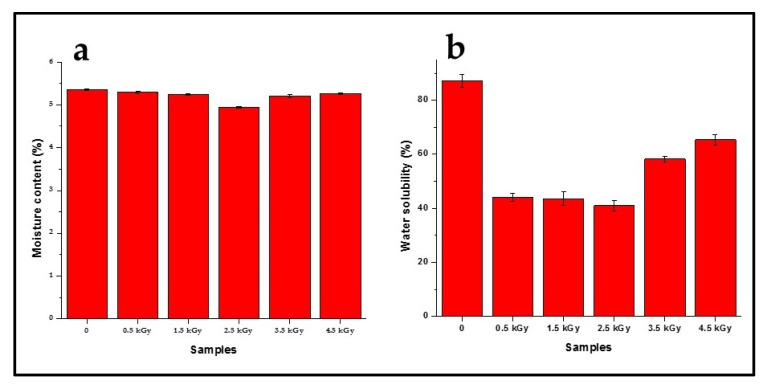
The (**a**) moisture content and (**b**) water solubility of seaweed-neem films at different doses of radiation.

The moisture content and water solubility (WS) of seaweed-neem films with different radiation doses were shown in Figure 3. The moisture content percentage decreased with increased irradiation doses. While the WS of the control film was very high (87.12% ± 2.26%), which was probably due to the hydrophilic nature of seaweed, moreover, the addition of hydrophilic glycerol as a plasticizer of the seaweed film contributed to the increase in the film solubility in water. Glycerol also weakened the interaction of seaweed polymer chains with open space between the chains. This, in turn, promoted water diffusion into the seaweed matrix and consequently resulted in increased solubility of plasticized seaweed film [31]. As shown in Figure 3, the irradiation doses at 0.5 to 2.5 kGy resulted in the reduction of WS, about 49% to 53%. A further increase of the irradiation doses (from 3.5 up to 4.5 kGy) on the seaweed-neem films resulted in the increase of WS to 58.23% ± 1.15% and 65.35% ± 1.91%, respectively.

**Figure 4:**
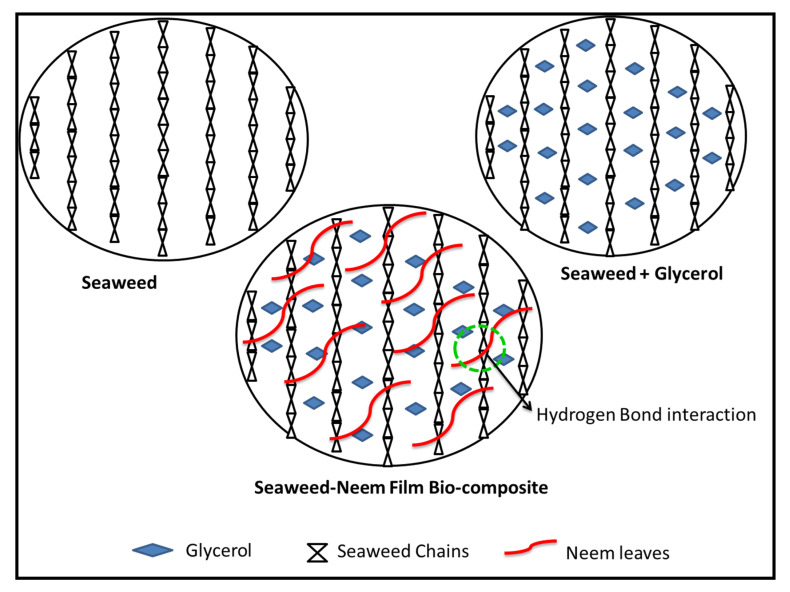
Schematic diagram of seaweed matrix interactions with neem leaves extract. The green circle represents hydrogen bond.

The decrease in moisture content and WS of the film with an increase in the radiation dose was due to the reduced hydrophilicity caused by the radiation and strong hydrogen bond formation. The hydrogen bond was formed between the hydroxyl groups in neem leaves molecules with hydroxyl groups in the seaweed film matrix that resulted in the enhanced stability of seaweed-neem films. A simplified illustration of seaweed matrix interactions with neem leaves extracts incorporated after the cast has been presented in Figure 4. The moisture content and water solubility values obtained corroborated the mechanical properties.

### 3.5. Water Vapour Permeability

**Figure 5:**
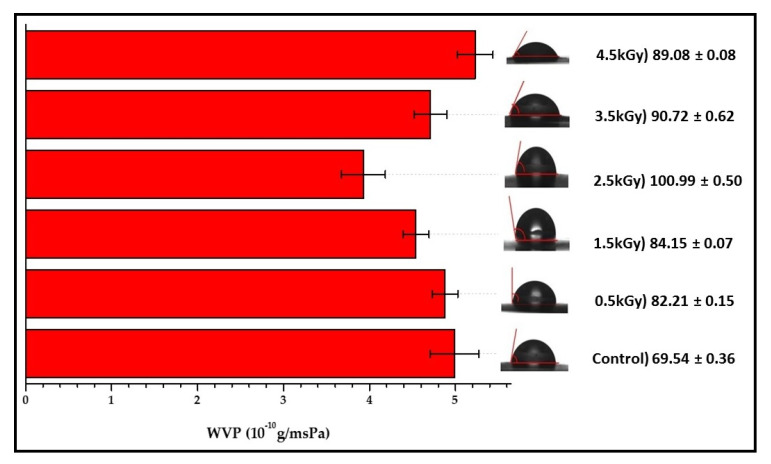
Water vapour permeability and contact angle of seaweed-neem films with different doses of radiation.

The water vapour permeability (*WVP*) values of the control and irradiated seaweed-neem films were displayed in Figure 5. The WVP value obtained for the control seaweed-neem film was 4.99 × 10^−10^ g/msPa. At the dose of 0.5, 1.0, and 2.5 kGy, irradiation produced a significant reduction of WVP from 4.88, 4.54 to 3.93 × 10^−10^ g/msPa. The WVP values were decreased with increased irradiation doses from 0.5 to 2.5 kGy. The seaweed-neem film irradiated with 2.5 kGy showed the lowest WVP value among the treatments. At 3.5 kGy, the film showed an increase in the WVP value, 4.71 × 10^−10^ g/msPa, and the WVP values increased as the doses of radiation increased up to 4.5 kGy. The seaweed-neem film irradiated with 4.5 kGy had the highest WVP compared with all the seaweed-neem films. Moreover, this decrease may be due to the enhanced hydrophobicity by irradiation. The enhanced hydrophobicity may be the reason behind the decrease and increase variations in the WVP values of the fabricated seaweed-neem films at the exposure of various radiation doses.

### 3.6. Contact Angle

The results obtained from the study of the contact angles of the seaweed-neem films were presented in Figure 5. The seaweed-based films have been reported with a hydrophilic nature, but the incorporation of 5% neem leaves extract into the seaweed matrix caused the control seaweed-neem film to exhibit a relatively high contact angle (69.54°). This neem leaves incorporation has resulted in the reduction of available hydroxyl groups [22,25,28]. Based on the obtained results, it was noted that the contact angles of the films increased with an increased radiation dose of up to 2.5 kGy.

However, at a radiation dose of 3.5 and 4.5 kGy, the water contact angles decreased from 90.72 to 89.08°, but the contact angle values were still higher than the control seaweed-neem film. The increase of contact angles indicated that the films’ hydrophobic nature increased.

**Figure 6:**
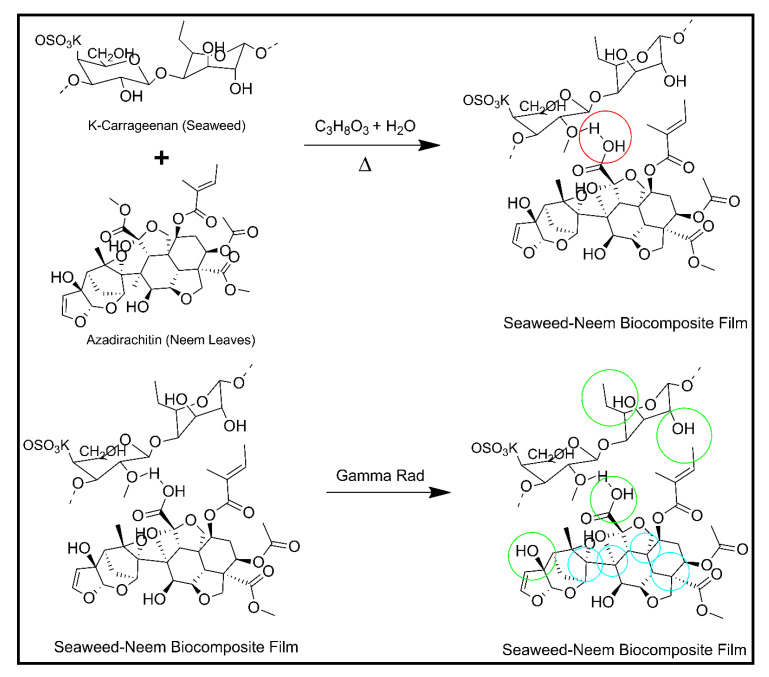
Proposed mechanism of seaweed-neem biocomposite film irradiated with gamma radiation. The blue circle represents C–Tertiary bond breakage, the green circle represents C–OH bond breakage and red circle represents H- bond.

The control film was characterized by an uneven surface or rough surface with a low contact angle (< 70°), while the irradiated films was characterized by a smooth surface with a higher contact angle (> 80°). The effect of irradiation on the hydrophobicity of the seaweed-neem films have rarely been studied. Soliman, Mohy Eldin [32] reported that the water contact angles of zein films increase with an increased radiation dosage up to 40 kGy. Figure 6 presented the mechanism of possible interactions of gamma irradiation on seaweed-neem film.

### 3.7. Mechanical Properties of Films

The mechanical properties of seaweed-based films incorporated with a 5% neem leave extract and various radiation doses were presented in Table 2. The seaweed-based film with a 5% neem leave extract (control group) had a tensile strength (TS) value of 39.95 MPa. The irradiation doses from 0.5 to 4.5 kGy on the control films increased the tensile strength of the films from 39.95 to 43.23 MPa. The tensile strength result showed that from 0.5 to 2.5 kGy, the TS values were increased with the increase in radiation dose. At doses of 0.5 and 2.5 kGy, the TS values of the irradiated films were 40.57 and 43.23 MPa, which were about 1.6% and 8.3% higher, respectively, compared with the control film. At doses higher than 2.5 kGy, the increase in irradiation doses decreased the TS values of the films significantly (*p* < 0.05). The enhancement in mechanical properties of seaweed-neem films at low irradiation doses (0.5–2.5 kGy) may be attributed to the enhanced hydrophobicity and the generation of free radicals, active sites in the composite seaweed film. However, at doses higher than 2.5 kGy, the TS values decreased significantly. The decrease in TS values at higher irradiation doses may be due to the degradation when seaweed was exposed to radiation. Seaweed, a polysaccharide, generally undergoes degradation under a higher dose of radiation. The results obtained in this study were similar to those reported by Gul-E-Noor, Khan [33].

The elongation at break of the seaweed-neem based film was shown in Table 2. The different doses of radiation (0.5 to 4.5 kGy) on the seaweed-neem films decreased the elongation at break (EAB) values from 19.39% to 17.54%. It was observed that the EAB values of seaweed-neem films after the radiation treatment were lower than the films’ EAB values before irradiation. At the same time, this value does not change significantly with increased irradiation doses; for example, 0.5 kGy EAB was found to be 19.25%. The EAB values decreased with an increase in the radiation dose and reached a minimum of 17.54% for 4.5 kGy. The decrease of the EAB values might be attributed to a possible radiation-induced degradation of seaweed components when the irradiation dose was increased. Therefore, the radiation up to a specific dosage induced improvement in the tensile strength and negligible decrease in elongation percentages of the seaweed-based neem leaves extract films. The application of radiation doses did not show a significant influence (at *p* > 0.05) on the film thickness. However, as the irradiation doses increased from 0.5 to 4.5 kGy, the seaweed-neem films were thicker (0.1072 to 0.1090 mm) compared to the film thickness of the film without irradiation (0.1060 mm). The values of the irradiated seaweed-neem were closer to the film without irradiation.

### 3.8. Biodegradability

**Figure 7:**
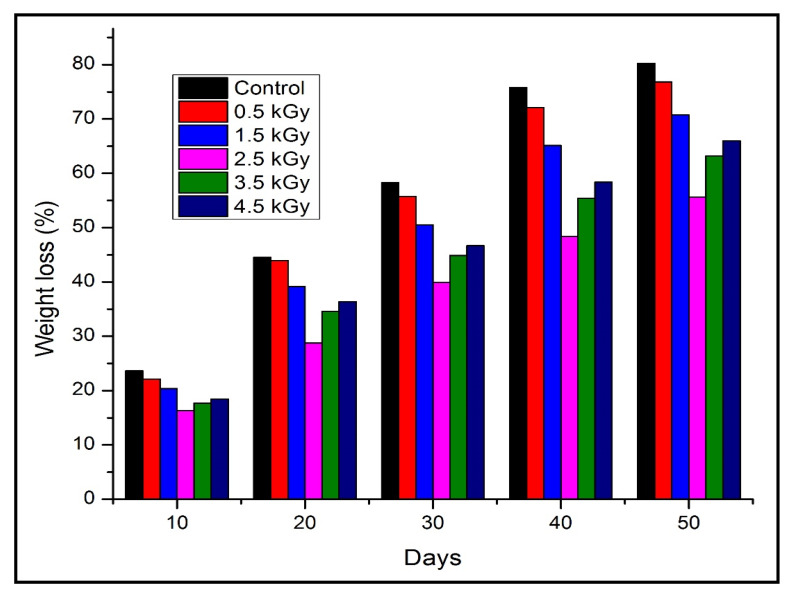
Weight loss of seaweed-neem composite films after soil burial for 10 to 50 days.

**Figure 8:**
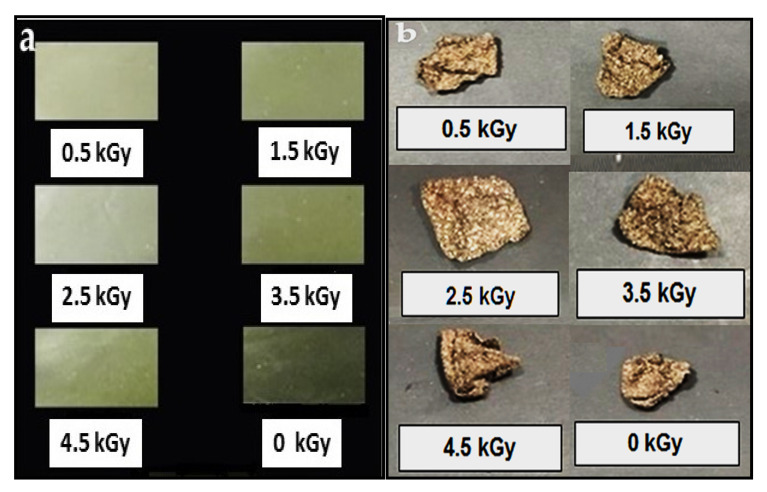
Digital image of the biodegradation test (**a**) before buried in soil and (**b**) after withdrawn from the soil after 50 days for non-irradiated and irradiated seaweed-neem composite films.

The weight of seaweed-neem composite films decreased with the number of days of burial. The biodegradation occurred even after 10 days of exposure in soil. Figure 7 showed the weight loss percentage of seaweed-neem films after soil burial for 10 to 50 days, respectively, and Figure 8 showed the digital image of the films after the biodegradability test. The control film showed the highest weight loss, at 80.8%, and the seaweed-neem composite film with a radiation dose of 2.5 kGy showed the lowest weight loss of 57.8% over the 50 days. This result indicated that the rates of biodegradation for control films were higher compared to the irradiated seaweed-neem film. These results were in agreement with Figure 7, where it was clearly shown that the degradation rate of the control film was higher compared to the irradiated seaweed-neem film. The control film was easily degraded because it was fabricated from seaweed and prone to a microorganism attack [34]. The application of radiation doses from 0.5 to 2.5 kGy on the seaweed-neem films appeared to slow down the rate of film biodegradation of seaweed-neem composite films due to the decomposition of bonds when exposed to irradiation. The radiation prevented the action of the microorganisms on the composite films, and the films were more resistant to degrade in soil. The results obtained from this study concurred with those reported by previous researchers [35]. In Figure 7, the weight loss of the buried samples was more significant between 30 to 50 days. The increase in the weight loss rate was attributed to an increase in microbial activities with the number of days [36]. The degradation caused by microorganism activities was due to the hydrophilic nature of neem leaves and seaweed with the presence of glycerol. The nature of the seaweed and neem leaves enhances the water absorption capability, which promotes microorganism growth and degrades seaweed-neem films, which reduces the weight of the films. Glycerol from the composite film diffused through the cell membrane and metabolized by the microorganisms that further enhanced the weight loss of the films [37]. Even though the biodegradability test of the composite films was done for 50 days, but the degradation process of the films did not stop after 50 days. Rather, it continued slowly until the seaweed-neem composite films were completely degraded.

### 3.9. Effect of Irradiation Dose on the Antimicrobial Activity of Seaweed-Neem Films

**Figure 9:**
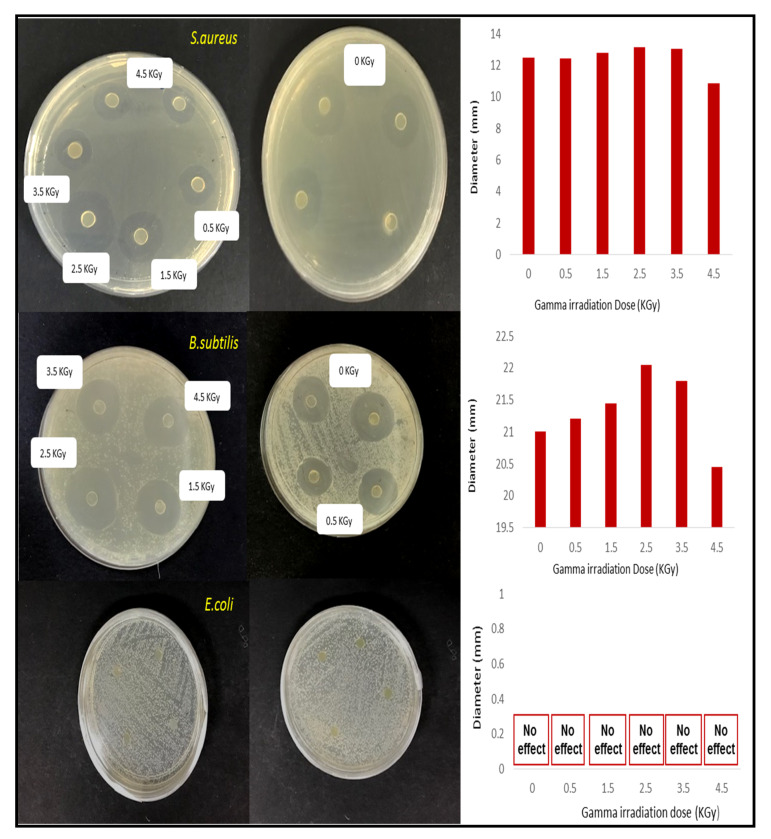
Inhibition zone measurement results (photographic images and diameter statistics of inhibition zone) of seaweed-neem composite films under the various radiation doses against *Staphylococcus aureus*, *Escherichia coli*, and *Bacillus subtilis*.

The antimicrobial of the seaweed-neem film with a radiation application has not been explored. The previous study on the antimicrobial activity of seaweed-neem film against food pathogenic bacteria showed that the seaweed-neem film had an excellent antimicrobial activity towards *Staphylococcus aureus* and *Bacillus subtilis* [22]. The antimicrobial activity of neem leaves extract-based seaweed films might be due to the presence of different bioactive constituents such as carotenoids, phenolic compounds, flavonoids, triterpenoids, ketones, valavinoids, saponins, glycosides, steroids, and tetra-triterpenoids azadirachtin in the neem leaves [38] because they were the principle antibiotics of the neem plant, used as a defensive mechanism against different pathogens. Inhibition zone measurements investigated the antimicrobial activity of seaweed-neem composite films with the application of various irradiation doses (0, 0.5, 1.5, 2.5, 3.5, and 4.5 KGy) against *Staphylococcus aureus, Escherichia coli*, and *Bacillus subtilis*, and the results were presented in Figure 9. The irradiated seaweed-neem films showed inhibitory zones against *Staphylococcus aureus* and *Bacillus subtilis*, but there was no appearance of inhibition zone observed for *Escherichia coli*. The results clearly showed that the antimicrobial activity of composite films against *Bacillus subtilis* was stronger than *Staphylococcus aureus*. The irradiated composite films showed the highest antimicrobial activities against *Bacillus subtilis* and *Staphylococcus aureus* at the dose of 2.5 KGy with the diameter of inhibition zone, 22.05 and 13.15 mm, respectively. As the irradiation dose increased from 3.5 to 4.5 KGy, the antimicrobial activities of the composite films on both *Bacillus subtilis* and *Staphylococcus aureus* decreased, as evidenced by the decrease of the inhibition zone diameters in Figure 9. It could be attributed that as the irradiation doses increased, the rupture of seaweed chains and the radical-induced by irradiation increased the amount of positive charge on seaweed chains, resulted in the enhanced antimicrobial activity. The decrease of zone inhibition at higher irradiation doses, 3.5 and 4.5 KGy, was caused by the decrease of positive charge amount on seaweed chain in the reaction between groups from neem leaves and seaweed molecules. In conclusion, the radiation treatment up to a dose of 2.5 KGy showed no significant (*p* < 0.05) change in antimicrobial activities of the seaweed-neem films against *Staphylococcus aureus* and *Bacillus subtilis* as the diameter of the inhibition zone was almost the same compared with the control film, 21 mm against *Bacillus subtilis,* and 12.5 mm against *Staphylococcus aureus.* This result was in good agreement with the previous study on a polyamide coated LDPE film with active compounds (sorbic acid, carvacrol, thymol, and rosemary oleoresin) where the antimicrobial activity of the films was found to get retained when exposed to 1–3 kGy [39].

## 4. Conclusions

This study successfully fabricated seaweed-based films incorporated with neem leaves extract and treated with gamma radiation. The films showed substantial improvement in the mechanical with excellent antimicrobial activity towards *Staphylococcus aureus* and *Bacillus subtilis,* which was highly desirable for the packaging application. The results also indicated that the irradiation doses up to 2.5 kGy on the biocomposite films showed reduced water vapour permeability, moisture content, and water solubility percentages. The results showed a substantial improvement in the barrier properties of the seaweed-neem composite films. The seaweed-neem films also showed an increase in film transparency up to a radiation dose of 2.5 kGy as consumers desire transparency, and it presented a good visual presentation for the packaged product. The morphology of the irradiated composite films displayed a smoother surface compared to the seaweed-neem film (control). Therefore, it resulted in the enhanced mechanical strength of the films with irradiation. Moreover, the seaweed-neem composite films with a radiation application resulted in the enhancement of the contact angle from 69.54° to 100.99° and increased the hydrophobicity of the films. The increased hydrophobicity of the seaweed-neem films was due to the oxidation process induced in the film by irradiation and thereby reduced the water vapour permeability of the control film. The soil burial test results showed that the irradiated seaweed-neem films had a lower biodegradation rate, and a lower weight loss percentage indicated the suitability of the fabricated film as packaging material. Overall, this study established that the irradiated seaweed-neem composite film could be used as an excellent packaging. The irradiation dose of 2.5 kGy on the seaweed-neem composite film showed the optimum improvement in the mechanical, barrier, biodegradability, and antimicrobial properties. Therefore, this fabricated biodegradable composite film was selected for further packaging application analysis.

## Figures and Tables

**Table 1:** The colour and opacity of irradiated and nonirradiated seaweed-neem films.

Parameter	Dose (kGy)					
	0.5	1.5	2.5	3.5	4.5	Control
L*	74.5 ± 0.02 ^a^	76.1 ± 0.03 ^b^	80.5 ± 0.01 ^f^	79.1 ± 0.02 ^e^	78.6 ± 0.06 ^d^	83.8 ± 0.04 ^g^
a*	−0.5 ± 0.01 ^b,c^	−0.6 ± 0.01 ^a,b^	−0.6 ± 0.10 ^a^	−0.4 ± 0.01 ^c^	−0.4 ± 0.01 ^c^	−0.4 ± 0.03 ^bc^
b*	35.1 ± 0.02 ^f^	33.2 ± 0.37 ^e^	29.1 ± 0.07 ^b^	30.4 ± 0.05 ^c^	30.6 ± 0.18 ^c,d^	21.5 ± 0.10 ^a^
∆*E*	116.3 ± 0.21 ^g^	114.3 ± 0.28 ^f^	109.0 ± 0.05 ^b^	110.7 ± 0.15 ^c^	113.4 ± 0.08 ^e^	104.3 ± 0.06 ^a^
Opacity	17.0 ± 0.02 ^c^	16.8 ± 0.03 ^b^	16.6 ± 0.01 ^a^	16.6 ± 0.04 ^a^	17.5 ± 0.03 ^d,c^	17.0 ± 0.01 ^c^

L* (lightness, 0 = black, 100 = white), a* (−a* = greenness, +a* = redness), and b* (−b* = blueness, +b* = yellowness). Values were presented as the mean ± standard deviation. Means in each column with different superscript letters (a–g) were significantly different (*p* < 0.05).

**Table 2:** The physical and mechanical properties of seaweed-neem composite films with different radiation doses.

Dose (kGy)	Thickness (mm)	Tensile Strength (MPa)	Tensile Modulus (MPa)	Elongation (%)
0.5	0.1072 ± 0.0013 ^a^	40.57 ± 0.67 ^c^	316.12 ± 18.70	19.25 ± 0.14 ^f^
1.5	0.1083 ± 0.0019 ^a^	41.81 ± 1.76 ^c^	335.37 ± 13.31	18.70 ± 0.14 ^e^
2.5	0.1085 ± 0.0058 ^a^	43.23 ± 0.25 ^d^	355.90 ± 10.98	18.22 ± 0.08 ^d^
3.5	0.1088 ± 0.0096 ^a^	38.55 ± 0.22 ^b^	323.94 ± 15.79	17.85 ± 0.10 ^c^
4.5	0.1090 ± 0.0083 ^a^	36.79 ± 0.73 ^a^	314.62 ± 17.17	17.54 ± 0.06 ^b^
Control	0.1060 ± 0.0053 ^a^	39.95 ± 0.35 ^c,d^	309.05 ± 10.57	19.39 ± 0.74 ^a,d^

Values were presented as the mean ± standard deviation. Means in each column with different superscript letters (a–f) were significantly different (*p* < 0.05).
